# Giant Prenatally Diagnosed Cervicofacial Cystic Lymphangioma in a Term Neonate: Case Report and Literature Review

**DOI:** 10.7759/cureus.95933

**Published:** 2025-11-02

**Authors:** Wijdane Lemcirdi, Hanae Bahari, Mohammed Ech-chebab, Sahar Messaoudi, Anass Ayyad, Rim Amrani

**Affiliations:** 1 Department of Pediatrics, Centre Hospitalier Universitaire Mohammed VI Oujda, Oujda, MAR; 2 Department of Pediatrics, Mohammed First University, Oujda, MAR; 3 Department of Neonatology and Neonatal Intensive Care, Centre Hospitalier Universitaire Mohammed VI Oujda, Oujda, MAR; 4 Department of Neonatology, Mother and Child Laboratory, Faculty of Medicine and Pharmacy, Mohammed First University, Oujda, MAR; 5 Department of Neonatology and Neonatal Resuscitation, Faculty of Medicine and Pharmacy, Mohammed First University, Oujda, MAR; 6 Department of Mother and Child Health Laboratory, Faculty of Medicine and Pharmacy, Mohammed First University, Oujda, MAR

**Keywords:** cervicofacial malformation, cystic lymphangioma, neonatal surgery, prenatal diagnosis, sclerotherapy

## Abstract

Cystic lymphangiomas are rare congenital malformations of the lymphatic system, most commonly affecting the cervicofacial region. Advances in prenatal imaging have improved antenatal detection, facilitating multidisciplinary coordination in perinatal care.

We report a term male neonate with a giant cervicofacial cystic lymphangioma diagnosed antenatally in the third trimester. Clinical examination revealed a large, transilluminable mass without immediate airway compromise. CT angiography demonstrated a multiloculated cystic lesion extending into the parapharyngeal and mediastinal spaces, closely related to major neurovascular structures. Complete surgical excision was performed during the neonatal period, with preservation of vital anatomy. Histopathology confirmed a benign venolymphatic malformation.

Optimal management of cervicofacial lymphangiomas relies on early recognition, precise imaging assessment, and coordinated multidisciplinary care.

Surgical excision is the mainstay of therapy, complemented by adjuvant modalities such as sclerotherapy, laser therapy, or pharmacological agents when indicated, and continuous surveillance is mandatory to prevent recurrence.

## Introduction

Cystic lymphangiomas are rare, benign congenital anomalies resulting from defective embryologic maturation or failure of connection between developing lymphatic vessels [1,2]. These lesions are classified as hamartomatous vascular malformations, characterized by localized dilatations and proliferations of lymphatic channels [3]. The cervicofacial area, particularly the posterior cervical triangle, is the most frequently affected site, accounting for nearly 75% of cases [3,4]. Most lesions are detected at birth or during early childhood; however, advances in prenatal ultrasonography and fetal MRI have significantly improved antenatal detection rates [2,5]. Their clinical importance lies in their potential to compress major cervical structures, including the carotid sheath, vagus, hypoglossal, and facial nerves, and to impinge on or narrow the airway and esophagus, thereby complicating diagnosis, prognosis, and surgical management [6]. 

Since Wernher’s first description in 1843, the term “cystic hygroma” has been historically used to describe what is now referred to as cystic lymphangioma or macrocystic lymphatic malformation [1]. We report a rare case of a giant cervicofacial cystic lymphangioma diagnosed prenatally in a term newborn, detailing the clinical, radiological, and surgical management and reviewing the relevant literature.

## Case presentation

We report the case of a male newborn referred to our neonatology unit for evaluation and management of a large left-sided cervicofacial mass, initially detected on obstetric ultrasound at 30 weeks of gestation during the third trimester. The mother, a 35-year-old gravida 3 para 2, had an otherwise uneventful pregnancy. She attended four scheduled prenatal visits, and aside from the cervical mass, no additional fetal anomalies were identified on prenatal assessment.

**Figure 1:**
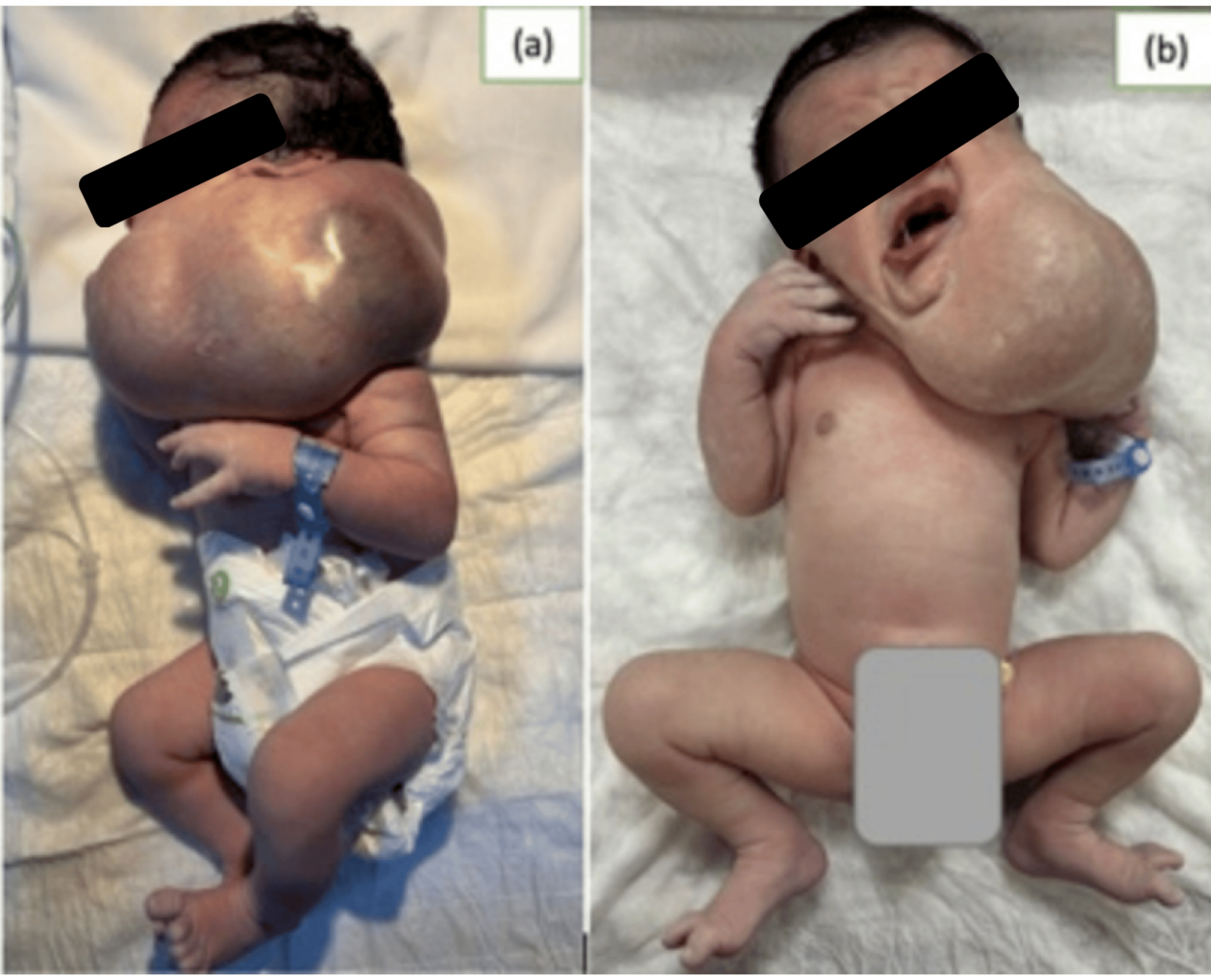
Clinical image of the newborn: (a) lateral and (b) frontal views demonstrating a large left cervicofacial mass.

The newborn was delivered at term by scheduled cesarean section, with a birth weight of 4500 g and Apgar scores of 10 at both the first and fifth minutes. At birth, the neonate was stable from both cardiovascular and respiratory standpoints. Examination revealed a soft, compressible, non-painful, transilluminable swelling in the left cervicofacial region, without skin discoloration. On auscultation, a systolic thrill could be appreciated (Figure 1).

Laboratory investigations showed normal hemoglobin level, white blood cell count, and prothrombin time, with negative alpha-fetoprotein.

**Figure 2:**
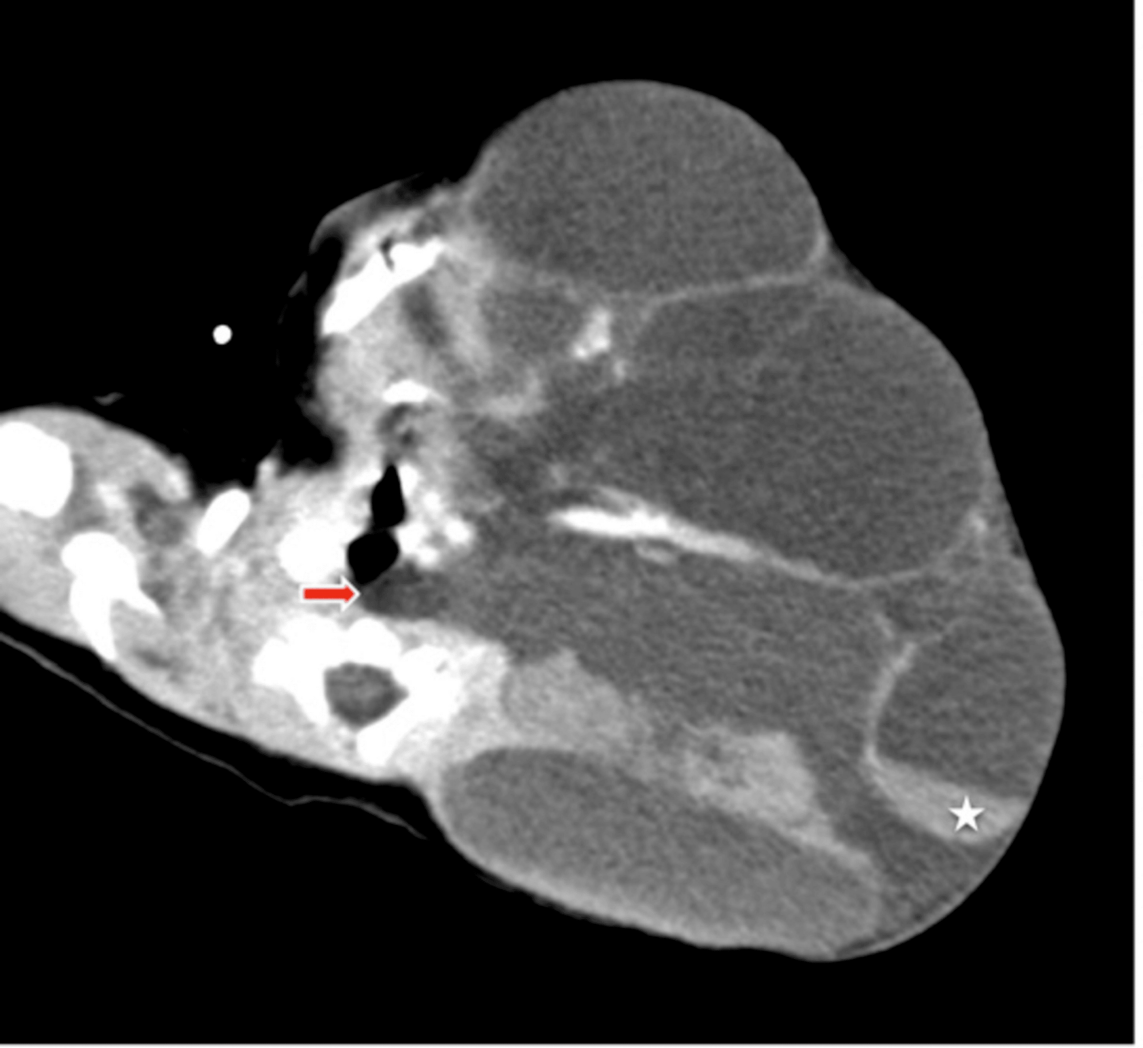
Contrast-enhanced CT image demonstrating a large multiloculated cystic mass in the left cervicofacial region, containing fluid and hemorrhagic components (asterisk). Several septa appear thickened, with internal extension toward the prevertebral space (arrow) and close contact with ipsilateral cervicofacial structures.

Contrast-enhanced CT angiography demonstrated a multiloculated, fluid-filled cystic formation with partial internal hemorrhage, measuring 147 × 98 mm. The lesion extended deeply into the prevertebral compartment and superior mediastinum, enveloping the internal jugular vein, carotid bifurcation, and structures at the skull base, without evidence of vascular occlusion. It also spread to the parapharyngeal, parotid, and masticator spaces, further reaching the submandibular and temporal regions (Figure 2).

Surgical removal was performed in the neonatal period on day 11 of life. Careful dissection allowed separation of the mass from the internal jugular vein, carotid artery, vagus nerve, cranial nerves (hypoglossal and lingual), and brachial plexus. The mass was excised completely while preserving vital neurovascular elements. 

**Figure 3:**
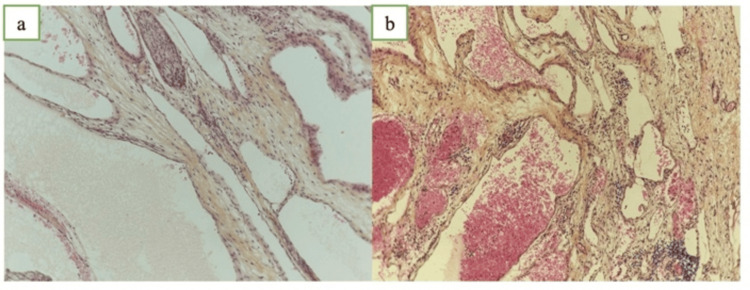
Fibrous connective tissue containing multiple ectatic structures filled with acellular intraluminal material (a, H&E ×200). Several vascular channels engorged with blood are also observed in certain areas (b, H&E ×200).

Histopathological analysis confirmed a benign venolymphatic malformation without malignant features (Figure 3). The examination was performed at the department of pathology, Mohammed VI University Hospital, Oujda, Morrocco.

The postoperative evolution was clinically satisfactory, with no immediate complications such as bleeding, infection, or airway compromise. The newborn showed favorable recovery, with progressive reduction of local swelling and satisfactory wound healing. Follow-up imaging (contrast-enhanced CT scan) confirmed the absence of residual mass or recurrence. At subsequent clinical evaluations, the patient remained stable, and the overall evolution was favorable. 

## Discussion

Lymphangiomas are rare malformations, with an estimated prevalence in the general population ranging between approximately 1 in 2,000 and 1 in 6,000 live births [7]. They are benign congenital anomalies of the lymphatic system, arising from defects in embryologic development, most often due to sequestration or abnormal connections between embryonic lymphatic channels [1,2]. While these malformations can theoretically develop in any anatomical region, the head and neck, particularly the posterior cervical triangle, are most frequently involved, representing nearly 75% of reported cases [3,4]. These lesions are typically characterized by multiloculated cystic spaces embedded within loose connective tissue, leading to a fluctuant, compressible mass [5].

While most lymphangiomas become apparent during infancy or early childhood, some can be diagnosed prenatally through advanced imaging, as in the case presented here. Although the cervicofacial region represents the most frequent location, uncommon sites have been documented, including the mediastinum, axilla, groin, oral cavity, and, more rarely, intra-abdominal organs such as the liver, spleen, or intestines, which together account for less than 5% of cases [6].

Regarding sex distribution, the literature presents varying data. De Serres et al. [4] reported a male-to-female ratio of approximately 1,2:1, indicating a slight male predominance in cervicofacial forms. Similarly, Kennedy et al. [1] documented a slight male predominance within their study population; however, this difference did not achieve statistical significance. Conversely, Triglia et al. [3] observed no significant sex difference in their pediatric population, suggesting that demographic and methodological factors may influence these variations. Collectively, these findings support a mild male bias without a strong sex-linked predilection.

Diagnosis is typically made based on clinical evaluation and imaging studies, although unusual presentations can delay recognition. In the neck region, approximately 85% of lymphangiomas occur on one side, with occasional spread to the same-side face or the floor of the mouth. Bilateral involvement or midline extension is less common, representing approximately 15% of cases [4]. These lesions may grow to significant proportions and, in certain cases, attain a massive size. In our case, the lesion presented as an extensive left-sided cervicofacial mass, extending from the neck into the parapharyngeal, parotid, masticator, submandibular, and temporal regions, with deep extension into the prevertebral space and superior mediastinum, encasing major neurovascular structures without causing vascular occlusion.

The clinical course of lymphangiomas is generally marked by slow, progressive growth. However, rapid expansion may occur due to secondary infections or hemorrhage within the lesion. Some cases have demonstrated spontaneous regression, though this is uncommon. Inflammatory exacerbations, often linked to nearby infections, may lead to transient swelling, and intralesional bleeding can result in sudden volume increases [8].

Ultrasound remains the standard imaging modality and is widely employed for the antenatal detection of cystic hygroma [8,9]. This technique provides valuable information regarding the lesion’s location, sonographic features, and any associated abnormalities. Additional imaging is often required to refine the diagnosis and evaluate the extent of the lesion to adjacent structures and vascular elements, particularly in anticipation of surgical management. Ultrasound serves as the first-line modality for defining both the location and the nature of the mass. The echotexture of cystic lymphangiomas typically resembles that of fluid-filled lesions, arranged in microcystic, macrocystic, or mixed patterns [10].

Computed tomography (CT) generally demonstrates a hypodense, well-circumscribed mass without evidence of invasion into neighboring anatomical structures. Magnetic resonance imaging (MRI) complements CT by offering superior visualization of the lesion’s relationships with surrounding tissues [11]. In our case, CT imaging was systematically performed.

The therapeutic management of cervicofacial lymphangiomas, particularly when lesions are extensive or symptomatic, requires a multidisciplinary approach. Surgical excision remains the gold standard, especially for large malformations involving the neck, oral cavity, or tongue, where compressive symptoms such as airway obstruction, stridor, or dysphagia frequently justify intervention [12-15], In our patient, although no compressive signs were present, surgical excision was performed during the neonatal period, allowing complete removal of the mass and preservation of vital structures.

Nonsurgical modalities have gained importance as alternative or adjunctive strategies. Sclerotherapy has shown efficacy, particularly in macrocystic lesions, with agents such as OK-432 (picibanil), bleomycin, doxycycline, and ethanol demonstrating favorable outcomes and minimal invasiveness [14]. Among these, intralesional bleomycin is widely employed for deeply infiltrating macrocystic malformations, while OK-432 has been successfully used both postnatally and prenatally, with intrauterine injections leading to significant lesion reduction without major complications [16].

In selected cases where airway compromise is anticipated at birth, the Ex Utero Intrapartum Treatment (EXIT) procedure may be performed. This approach maintains placental circulation during delivery, enabling secure airway management prior to complete separation from maternal circulation, a critical strategy for neonates with massive cervicofacial lymphangiomas [17].

Microcystic and cavernous forms often require combined treatment modalities. Okazaki et al. recommend an initial course of OK-432 followed by surgical excision for improved outcomes [18]. Additional interventions such as CO₂ or Nd:YAG laser therapy, cryotherapy, radiofrequency ablation, or aspiration may be considered in refractory or selected cases [13,14]

Beyond established treatments, novel therapies are under investigation. Agents such as sirolimus, propranolol, and sildenafil, along with surgical innovations like vascularized lymph node transfer, are emerging as promising options in complex or recurrent cases [19].

In our case, the patient was managed exclusively by surgical excision, resulting in an estimated remission rate of 82%. These results are in line with published data, as Flanagan reported a surgical success rate of 75%, with long-term follow-up beyond five years in 74% of patients, while Hancock observed an overall remission rate of 77.4%, with 52.5% of tumors located in the head and neck. Similarly, Alqahtani et al. documented complete resolution in 77% of patients, including 34.9% with cervicofacial involvement [20-22].

Despite their benign histology, lymphangiomas may cause significant morbidity due to local invasion, compression, or recurrence. Reported recurrence rates after surgery range from 5-15% in cases of complete excision but can reach nearly 100% following incomplete resection [13,23,24]. For this reason, long-term follow-up is mandatory. In our case, complete excision was achieved during a single surgical procedure without compromise of vital structures, and no recurrence has been observed to date after an 18-month follow-up period.

## Conclusions

Giant cervicofacial cystic lymphangiomas are rare congenital malformations that pose major diagnostic and therapeutic challenges in the neonatal period. Advances in prenatal imaging have improved early recognition and multidisciplinary planning. Complete surgical excision remains the cornerstone of treatment for extensive or symptomatic lesions, whereas sclerotherapy and other minimally invasive techniques may be considered in selected cases. Long-term follow-up is essential to monitor recurrence and ensure optimal outcomes.
